# Estimating similarity and distance using FracMinHash

**DOI:** 10.1186/s13015-025-00276-8

**Published:** 2025-05-15

**Authors:** Mahmudur Rahman Hera, David Koslicki

**Affiliations:** 1 School of Electrical Engineering and Computer Science, Pennsylvania State University, University Park, USA; 2 Huck Institutes of the Life Sciences, Pennsylvania State University, University Park, USA; 3 Department of Biology, Pennsylvania State University, University Park , USA

**Keywords:** Hashing, Sketching, FracMinHash, Min-Hash, k-mer, Similarity, Theory

## Abstract

**Motivation:**

The increasing number and volume of genomic and metagenomic data necessitates scalable and robust computational models for precise analysis. Sketching techniques utilizing $k$-mers from a biological sample have proven to be useful for large-scale analyses. In recent years, FracMinHash has emerged as a popular sketching technique and has been used in several useful applications. Recent studies on FracMinHash proved unbiased estimators for the containment and Jaccard indices. However, theoretical investigations for other metrics are still lacking.

**Theoretical contributions:**

In this paper, we present a theoretical framework for estimating similarity/distance metrics by using FracMinHash sketches, when the metric is expressible in a certain form. We establish conditions under which such an estimation is sound and recommend a minimum scale factor *s* for accurate results. Experimental evidence supports our theoretical findings.

**Practical contributions:**

We also present frac-kmc, a fast and efficient FracMinHash sketch generator program. frac-kmc is the fastest known FracMinHash sketch generator, delivering accurate and precise results for cosine similarity estimation on real data. frac-kmc is also the first parallel tool for this task, allowing for speeding up sketch generation using multiple CPU cores – an option lacking in existing serialized tools. We show that by computing FracMinHash sketches using frac-kmc, we can estimate pairwise similarity speedily and accurately on real data. frac-kmc is freely available here: https://github.com/KoslickiLab/frac-kmc/

## Introduction

With the growing number of reference genomes and the exponential increase in genomic and metagenomic data production, there is a critical need for the development of computational models that are both scalable and robust, as well as ensure precision in analysis. $k$-mer-based algorithms, particularly those utilizing sketching methods, are becoming increasingly popular for large-scale sequence analysis and metagenomic applications. A $k$-mer is a sequence of *k* consecutive nucleotides extracted from a longer sequence. Algorithms designed to work with $k$-mers decompose a long sequence into small *k*-mers and analyze based on the number of shared or dissimilar $k$-mers among multiple samples. Given the potentially vast number of distinct $k$-mers in a sequencing sample, sketching methods create a fingerprint of the $k$-mers (called a sketch) to work with these smaller sets, thereby reducing computational resource consumption. Using sketches become particularly useful when it is important to compare many query samples against many reference samples: as the sketches are much smaller, each of these many-vs-many computations become cheaper and more lightweight.

The most widely used sketching method for many years has been MinHash [5], originally introduced for document comparisons. Mash [31] was developed to apply MinHash to genomic data and has been extensively utilized. However, studies have revealed that MinHash sketches perform relatively poorly when comparing sets of very dissimilar sizes [5, 23, 25]. Researchers have proposed various adjustments to MinHash to address this issue [3, 21, 23, 30]. One such example is the recently introduced FracMinHash sketch, which uses a variable sketch size instead of MinHash’s fixed-size scheme. FracMinHash was first introduced and used in the software sourmash [6, 33]. In simple words, a FracMinHash sketch retains *s* ($0 \le s \le 1$) fraction of the input set of $k$-mers. The scale factor *s* is a tunable parameter of the FracMinHash sketching technique, controlling the size of the generated sketch.

The first theoretical analysis of FracMinHash was introduced in [18], which showed how to obtain an unbiased estimator of the containment and the Jaccard indices computed using FracMinHash sketches. This work laid the theoretical foundation for calculating average nucleotide identity (ANI) via FracMinHash sketches and led to useful applications, such as ANI estimation in metagenomes [39], obtaining taxonomy from metagenome samples [19], obtaining a functional profile from metagenomes [17], etc. Besides the Jaccard and the containment indices, there are other metrics used in the literature when comparing two samples, such as cosine similarity, Bray-Curtis dissimilarity, (first and second) Kulczynski measure, etc. A list of such metrics is given in Table 1. Aside from the containment and the Jaccard indices, cosine similarity is more widely used than the other metrics and has been used in finding similarities between chromosomes, genes, cell structures, and functions, and in many other applications [7, 8, 34, 41]. Although less common, the other metrics can also be useful in genomics as well as other disciplines such as ecology and community studies [4, 9, 10, 14, 35, 38].

As it has been around for many years, MinHash and its generalizations have been extensively studied from a theoretical point of view [5, 23, 24, 26, 40]. FracMinHash, on the other hand, has been introduced fairly recently, and a comprehensive analysis of various similarity/distance metrics in the context of FracMinHash is still missing. In this paper, we present this theoretical analysis for a class of similarity and distance metrics (which has been estimated using FracMinHash sketches). Let a similarity/distance measure between two non-empty sets *A* and *B* be $\mathscr {D}(A,B)$. Also, let $\mathscr {D}(A,B)$ be expressible as a certain form (which we introduce later in the paper). In this work, we show that there exists a scale factor *s*, for which, the metric $\mathscr {D}(A,B)$ can be accurately measured by $\mathscr {D}(\textbf{FRAC}_s(A), \textbf{FRAC}_s(B))$. We next show specific instances of $\mathscr {D}(A,B)$ in the form of the cosine similarity, the Bray-Curtis dissimilarity, the first Kulczynski measure, and the Sorensen index – and elaborate the required theoretical conditions. We supplement our theoretical findings with experimental evidence using simulations. These experiments show that our theoretical analyses are sound.

Apart from these theoretical results, our other contribution presented in this paper is implementing a fast, efficient, and parallel FracMinHash sketch generator program, frac-kmc. Although FracMinHash sketches can readily be generated using the software sourmash, we found the program sourmash sketch to be slow for very large samples. Furthermore, sourmash processes the $k$-mers in an input file in a serialized manner, and cannot parallelize the computation of a FracMinHash sketch. Therefore, we developed frac-kmc by modifying a $k$-mer-counter tool KMC [12, 13, 22]. To the best of our knowledge, frac-kmc is the fastest FracMinHash sketch generator program. Our results show that frac-kmc on a single thread is already nearly 70% faster than sourmash sketch, and frac-kmc can speed up FracMinHash sketch generation even more by using multiple CPU cores, an option lacking in sourmash. We used frac-kmc to compute FracMinHash sketches and used the sketches to estimate cosine similarity values on real data, and found accurate and precise results. frac-kmc is freely available here: github.com/KoslickiLab/frac-kmc. The analyses presented in this paper can be reproduced using the code https://www.github.com/KoslickiLab/fmh_cosine_reproducibles.

## Preliminaries

We present the following preliminaries in their full generality, using generic notation such as $\Omega$, a universal set. All theorems presented in Section Theoretical Results also hold for any universal set. In the case of sequence comparisons, the sets of interest, *A* and *B* are sets of *k*-mers, in the universe $\Omega = \{A,C,G,T\}^k$.

**Family of strongly-universal hash functions** We define a range $\mathcal {R} = [1, H]$ as a set of integers from 1 to *H*. Given a universe $\Omega$ and a range $\mathcal {R}$, a hash function $h~:~\Omega \rightarrow ~\mathcal {R}$ maps elements in $\Omega$ to the range $\mathcal {R}$. In this work, we consider $\mathcal {H}$ as a family of strongly 2-universal (also known as 2-wise independent) hash functions^1^. A hash family $\mathcal {H}$ is strongly 2-universal if for every $e_i, e_j \in \Omega$, $e_i \ne e_j$, and for every $r_1, r_2 \in \mathcal {R}$, the following holds

$$\Pr _{h \sim \mathcal {H}} \Big [ h(e_i) = r _1 \text {~and~} h(e_j) = r _2 \Big ] = \frac{1}{ {\vert \mathcal {R} \vert }^2 }~~.$$

In simpler and more relaxed terms, if *h* is drawn uniformly randomly from $\mathcal {H}$, the following two hold:

1. Uniformity: for any fixed $e \in \Omega$, *h*(*e*) is uniformly distributed in $\mathcal {R}$
2. Independence: given any two fixed distinct elements $e_i, e_j \in \Omega$, $h(e_i)$ and $h(e_j)$ can be construed as independent random variates in $\mathcal {R}$

**FracMinHash sketching** Given a hash function *h* randomly drawn from $\mathcal {H}$ for some fixed $H\in \mathbb {N}$ where $H>> \vert ~ \Omega ~ \vert$, and given a fixed *scale factor* *s* where $0 \le s \le 1$, a FracMinHash sketch of a set *A*, where $A \subseteq \Omega$, is defined as follows:

1 $$\begin{aligned} \textbf{FRAC}_s(A) = \left\{ \,h(a) \mid a \in A\ \textrm{and}\ h(a) \le Hs\right\} . \end{aligned}$$

The scale factor *s* is a tuneable parameter that can modify the size of the sketch. If one sets $s = 0$, then $\textbf{FRAC}_s(A)$ results in an empty set; if one sets $s = 1$, then $\textbf{FRAC}_s(A)$ contains all the elements of *A* (or, more precisely, their hash-values). On the other hand, for a fixed *s*, if the set *A* grows larger, the sketch $\textbf{FRAC}_s(A)$ grows proportionally in size.

It is important to note that in practice, most FracMinHash implementations (sourmash and frac-kmc) use MurMurHash3 as the hash function. MurMurHash functions do not have a guarantee of universality, let alone strong universality. Yet, practical implementations yield results conforming to the theory we develop here assuming a strongly universal hash family.

**Difference with sketching using permutations**

Hash functions are frequently used to permute elements in a set. The permutations can, in turn, be used to compute a sample from a given set. Such sampling/sketching techniques include MinHash [5], bottom-*k* (top-*k*) sketching [11], and more recently introduced Affirmative Sampling [27], which takes advantage of the “hiring problem” [1] and various strategies used to solve the hiring problem (hiring above a certain quantile/rank) [15, 16].

To highlight the difference with such rank-based sketching algorithms, we note that $\textbf{FRAC}_s(A)$ is *not* defined on the permutation of $\Omega$ achieved by applying *h*, rather by using the hash-values themselves. This means that acceptance of an element *e* in the sketch does not depend on its rank, rather only on its hash value *h*(*e*) (and, of course, the acceptance threshold *Hs*).

**Similarity and distance measures between two sets** The degree of similarity and/or dissimilarity between two sets can be measured using several metrics. These metrics, or more precisely, similarity and dissimilarity measures, have different uses and interpretations, depending on the domain knowledge. Table 1 shows a number of these metrics as well as the mathematical expressions.

**Table 1:** Mathematical expressions for several similarity and dissimilarity measures

Metric name	Notation	Expression
Jaccard similarity	*J*(*A*, *B*)	$\frac{|A \cap B|}{|A \cup B|}$
Containment index	*C*(*A*, *B*)	$\frac{|A \cap B|}{|A|}$
Cosine similarity (also known as Otsuka-Ochiai)	$\cos \theta$	$\frac{|A \cap B|}{\sqrt{|A|\cdot |B|}}$
Kulczynski 1	$K_1(A,B)$	$\frac{|A \cap B|}{|A \Delta B|}$
Kulczynski 2	$K_2(A,B)$	$\frac{1}{2} \Big ( \frac{|A \cap B|}{|A|} + \frac{|A \cap B|}{|B|} \Big )$
Whittaker distance	*W*(*A*, *B*)	$1 - \frac{1}{2} \Big ( \frac{|A \cap B|}{|A|} + \frac{|A \cap B|}{|B|} \Big )$
Sorensen index	*S*(*A*, *B*)	$2 \frac{|A \cap B|}{|A| + |B|}$
Bray-Curtis dissimilarity	*BC*(*A*, *B*)	$1 - 2 \frac{|A \cap B|}{|A| + |B|}$

**Chernoff bound for sum of Bernoulli random variables** Recall the classic Chernoff bounds: Let $X_i$, $i = 1, 2,$ ..., *n* be *n* independent Bernoulli random variables. If $X = \sum _{i=1}^n X_i$ and $E[X] = \mu$, then the following holds for $0< \epsilon < 1$ [29]:

$$\Pr \Bigg [ \Big \vert X - \mu \Big \vert ~ \ge \epsilon ~ \mu \Bigg ] \le 2 \exp \Big \{-\epsilon ^2 \mu / 3\Big \}.$$

## Theoretical Results

Let $\mathscr {D}= \mathscr {D}\Big (A,B\Big )$ be a similarity/distance measure between two sets *A* and *B*, and let $\mathscr {D}' = \mathscr {D}\Big (\textbf{FRAC}_{s}(A),\textbf{FRAC}_{s}(B)\Big )$ be the same measure between the sketches of *A* and *B*. Ideally, we want $\mathscr {D}'$ to be an unbiased estimator of $\mathscr {D}$. Which is, we want $E[\mathscr {D}'] = \mathscr {D}$. Previous works have shown how to obtain unbiased estimators for the Jaccard index and the containment index [18, 19]. We notice that the second Kulczynski index and the Whittaker distance can be expressed as linear combinations of the two containment indices *C*(*A*, *B*) and *C*(*B*, *A*). Therefore, it is easy to obtain unbiased estimators for the second Kulczynski index and the Whittaker distance. These estimators are listed in Table 2.

**Table 2:** Unbiased estimators for the Jaccard similarity, the containment index, the second Kulczynski index, and the Whittaker distance, when using FracMinHash sketches instead of the original sets

Metric name	Expression	Unbiased estimator
Jaccard similarity	$J(A,B) = \frac{|A \cap B|}{|A \cup B|}$	$\hat{J}(A,B) = J\Big ( \textbf{FRAC}_{s}(A), \textbf{FRAC}_{s}(B) \Big ) \times \frac{1}{ 1 - (1-s)^{|A \cup B|} }$
Containment index	$C(A,B) = \frac{|A \cap B|}{|A|}$	$\hat{C}(A,B) = C\Big ( \textbf{FRAC}_{s}(A), \textbf{FRAC}_{s}(B) \Big ) \times \frac{1}{ 1 - (1-s)^{|A|} }$
Kulczynski 2	$K_2(A,B) = \frac{1}{2} \Big ( \frac{|A \cap B|}{|A|} + \frac{|A \cap B|}{|B|} \Big )$	$\hat{K_2}(A,B) = \frac{1}{2}\Big (\hat{C}(A,B) + \hat{C}(B,A)\Big )$
Whittaker distance	$W(A,B) = 1 - \frac{1}{2} \Big ( \frac{|A \cap B|}{|A|} + \frac{|A \cap B|}{|B|} \Big )$	$\hat{W}(A,B) = 1 - \hat{K_2}(A,B)$

For the other metrics listed in Table 1, proving an unbiased estimator is not mathematically tractable. For these metrics, we attempt to prove that the following holds with high probability:

2 $$\begin{aligned} \Big \vert \mathscr {D}' - \mathscr {D}\Big \vert \le ~ \epsilon ~ \mathscr {D}, \end{aligned}$$

for any arbitrarily small $\epsilon$. In other words, the similarity/distance measure between two FracMinHash sketches approximates the similarity/distance measure between the original sets. Unfortunately, this does not hold in all cases. In this section, we present theoretical conditions where Equation 2 holds (and where it breaks down) when $\mathscr {D}$ is expressible to a certain mathematical form. For the sake of continuity, all proofs of the theorems are included in Section Methods.

### Theorem 1

Let $\Omega = \{~e_i~\}_{i=1}^N$ be a given set (universe), and let $A \subseteq \Omega$. Let $\textbf{FRAC}_{s}(A)$ be the FracMinHash sketch of *A* for a given *s* where $0 \le s \le 1$, and let the cardinality of $\textbf{FRAC}_{s}(A)$ be $X_A$. The expected number of elements in $\textbf{FRAC}_{s}(A)$ is given by the following:

3 $$\begin{aligned} E\Big [ X_A \Big ] = ~s ~ \vert A \vert . \end{aligned}$$

### Proof

See Section Proofs of theorems. $\square$

Theorem 1 quantifies the expected number of elements in $\textbf{FRAC}_{s}(A)$. We next show that the number of elements in $\textbf{FRAC}_{s}(A)$ is well concentrated around this expected value.

### Theorem 2

Let $\Omega = \{~e_i~\}_{i=1}^N$ be a given set (universe), and let $A \subseteq \Omega$. If $\textbf{FRAC}_{s}(A)$ is the FracMinHash sketch of *A* for a given *s* where $0 \le s \le 1$, and if the cardinality of $\textbf{FRAC}_{s}(A)$ is $X_A$, then the following holds for any $\epsilon$ where $0< \epsilon < 1$.

4 $$\begin{aligned} \Pr \Bigg [ \Big \vert X_A - s ~ |A| ~\Big | \ge \epsilon ~ s ~ |A| ~\Bigg ] \le 2 \exp { \Big ( - s ~ |A| ~ \epsilon ^2/3 \Big ) }. \end{aligned}$$

### Proof

See Section Proofs of theorems. $\square$

We use the results in Theorems 1 and 2 to quantify the error in estimating specific instances of $\mathscr {D}$. We explicitly show this for the cosine similarity in the next three sections: Sections Safety conditions to estimate cosine similarity using FracMinHash, Recommended scale factor s to safely estimate the cosine similarity 189 using FracMinHash, and Theoretical conditions for the other metrics.

### Safety conditions to estimate cosine similarity using FracMinHash

Given two sets *A* and *B*, we can use the expected cardinality of $\textbf{FRAC}_{s}(A)$, $\textbf{FRAC}_{s}(B)$ and $\textbf{FRAC}_{s}(A) \cap \textbf{FRAC}_{s}(B)$ to prove the following result.

#### Theorem 3

Let $\Omega = \{~e_i~\}_{i=1}^N$ be a given set (universe), and let $A, B \subseteq \Omega$ be two sets in the universe. Let the cosine similarity of the sets *A* and *B* be $\cos \theta$, and that of the sets $\textbf{FRAC}_{s}(A)$ and $\textbf{FRAC}_{s}(B)$ be $\cos \theta '$, where $\textbf{FRAC}_{s}(A)$ and $\textbf{FRAC}_{s}(B)$ are the FracMinHash sketches of *A* and *B* respectively for a given *s* where $0 \le s \le 1$. Then, there exists a small $\epsilon$ where $0< \epsilon < 1$, such that the following holds

5 $$\begin{aligned} \Big \vert \cos \theta ' - \cos \theta \Big \vert \le \epsilon ~ \cos \theta \end{aligned}$$

with a probability of at least $1 - 6 \exp \Big \{-s ~ |~A\cap B~| ~\epsilon ^2 / ~[3(2+\epsilon )^2]\Big \}$.

#### Proof

See Section Proofs of theorems. $\square$

Theorem 3 indicates that the cosine similarity between two sketched sets approximates the cosine similarity of the original sets with approximation error being bounded by the relative similarity of the original sets.

We note that when *A* and *B* are highly dissimilar and the set $A \cap B$ is very small, the probability guarantee becomes less meaningful and cannot be interpreted as “high probability”. In such a case, $\textbf{FRAC}_{s}(A)$ and $\textbf{FRAC}_{s}(B)$ will have nearly zero elements, and $\cos \theta '$ will be close to zero. This small $\cos \theta '$ reflects the dissimilarity of *A* and *B*, and estimating cosine seems reasonable. Nevertheless, Theorem 3 cannot guarantee with high probability that two near-zero quantities ($\cos \theta '$ and $\cos \theta$) are sufficiently close to each other.

### Recommended scale factor *s* to safely estimate the cosine similarity using FracMinHash

We conclude our theoretical results for the cosine similarity by suggesting a minimum scale factor that is safe to use when estimating the cosine similarity using FracMinHash sketches. The probability guarantee in Theorem 3 allows us to recommend a scale factor *s* for a desired error rate $\epsilon$ and a desired level of confidence $\alpha$, $0 \le \alpha < 1$. We define the desired confidence level $\alpha$ as the minimum guarantee we wish to have on the tolerable error rate $\epsilon$ such that $\cos \theta ' \in (1 \pm \epsilon ) \cos \theta$.

If we want to have a guarantee of at least $\alpha$, $0 \le \alpha < 1$, that the estimated cosine $\cos \theta '$ will be in a $1\pm \epsilon$ factor of the true cosine $\cos \theta$ where $0 \le \epsilon < 1$, then we require a scale factor *s*, such that

6 $$\begin{aligned} s \ge \frac{3 (2+\epsilon )^2~ \ln \Big [ 6/(1-\alpha ) \Big ]}{\epsilon ^2 ~ \min \Big \{ |A|, |B|, |~A \cap B~| \Big \}}. \end{aligned}$$

If we want a higher level of confidence, or if we want a smaller window of error, we require a larger scale factor. If *A* and *B* have a large number of common elements, then a smaller *s* suffices. Since estimating cosine is reasonable for highly dissimilar pairs of sets, we found that recommending a scale factor by using ${\min \Big \{ |A|, |B| \Big \}}$ suffices for practical purposes. Therefore, we recommend a scale factor as follows:

7 $$\begin{aligned} s \ge \frac{3 (2+\epsilon )^2~ \ln \Big [ 6/(1-\alpha ) \Big ]}{\epsilon ^2 ~ \min \Big \{ |A|, |B|\Big \}}. \end{aligned}$$

**Table 3:** Conditions when estimating the cosine similarity, the Bray-Curtis dissimilarity, the first Kulczynski measure, and the Sorensen index (by using FracMinHash sketches) are theoretically sound. These are shown for $0 \le \epsilon < 1.$

Metric name	Error bound	Probability with which error is bounded
Cosine similarity	$\Big \vert \cos \theta ' - \cos \theta \Big \vert \le \epsilon ~ \cos \theta$	$>= 1 - 6 e^{-s ~ |~A\cap B~| ~\epsilon ^2 / ~[3(2+\epsilon )^2]}$
Kulczynski 1	$\Big \vert {K_1}'(A,B) - {K_1}(A,B) \Big \vert \le \epsilon ~ {K_1}(A,B)$	$>= 1 - 4 e ^ {-s ~ \min \{|A\cap B|,|A\Delta B|\} ~\epsilon ^2 / ~[3(2+\epsilon )^2]}$
Sorensen index	$\Big \vert S'(A,B) - S(A,B) \Big \vert \le \epsilon ~ S(A,B)$	$>= 1 - 6 e^{-s ~ |~A\cap B~| ~\epsilon ^2 / ~[3(2+\epsilon )^2]}$
Bray-Curtis dissimilarity	$\Big \vert BC'(A,B) - BC(A,B) \Big \vert \le \epsilon ~ BC(A,B)$	$>= 1 - 6 e^{-s ~ |~A\cap B~| ~\epsilon ^2 / ~[3(2+\epsilon )^2]}$

### Theoretical conditions for the other metrics

We conclude the theoretical results section by recognizing that similar theoretical conditions can be derived for the other metrics in Table 1 (for which, an unbiased estimator cannot be proven). These theoretical conditions can be manipulated to obtain a recommended minimum scale factor for each of these metrics. For the sake of brevity, we show these conditions in Table 3 instead of writing individual theorems such as Theorem 3. For the entirety of Table 3, we use the notation that $X_S = | ~ \textbf{FRAC}_{s}(S) ~ |$ for some set *S*.

## Experimental Results

**Table 4:** Suggested scale factors for various levels of desired confidence and various tolerable rates of error, when $\min (m,n) = 10000$. For only 10K elements, if the tolerable error is up to 7%, we cannot but use all elements to get the desired accuracy

	Desired level of confidence, $\alpha$				
Tolerable Error, $\delta$	0.91	0.93	0.95	0.97	0.99
0.01	1.0000	1.0000	1.0000	1.0000	1.0000
0.03	1.0000	1.0000	1.0000	1.0000	1.0000
0.05	1.0000	1.0000	1.0000	1.0000	1.0000
0.07	1.0000	1.0000	1.0000	1.0000	1.0000
0.09	0.6794	0.7201	0.7745	0.8572	1.0000
0.1	0.5556	0.5889	0.6334	0.7010	0.8463

In this section, we present our experimental results. We first show results supporting the theory we presented in Section Theoretical Results. Then, we discuss a fast and efficient program to compute FracMinHash sketches from nucleotide sequences. We named this program frac-kmc. Finally, we present the performance of frac-kmc on real biological sequences. For the sake of brevity, we only show results for the cosine similarity and the Bray-Curtis dissimilarity in this section, although we can obtain similar results for any of the similarity/distance metrics listed in Table 3.

All results presented in this section have been generated on a server computer; having two AMD EPYC 7763 Processors. It has 128 physical cores, distributed across two sockets, and supports SMT (two simultaneous threads per core); allowing for up to 256 logical cores. The processor has 4 MiB L1 instruction caches (128 instances), 4 MiB L1 data caches (128 instances), 64 MiB L2 caches (128 instances), and 512 MiB L3 caches (16 instances). The processor can operate at a maximum clock-speed of 3.53 GHz. The computer has 3.9 TiB of main memory and has 60 TBs of SSD disk storage.

### Our suggested scale factors are safer to estimate the cosine similarity using FracMinHash sketches

**Table 5:** Suggested scale factors for various levels of desired confidence and various tolerable rates of error, when $\min (m,n) = 10000000$. For 10M elements, we can use a small fraction of the elements to get the desired accuracy when estimating the cosine similarity

	Desired level of confidence, $\alpha$				
Tolerable Error, $\delta$	0.91	0.93	0.95	0.97	0.99
0.01	0.0509	0.0539	0.0580	0.0642	0.0775
0.03	0.0058	0.0061	0.0066	0.0073	0.0088
0.05	0.0021	0.0022	0.0024	0.0027	0.0032
0.07	0.0011	0.0012	0.0013	0.0014	0.0017
0.09	0.0007	0.0007	0.0008	0.0009	0.0010
0.1	0.0006	0.0006	0.0006	0.0007	0.0008

We start by presenting what the scale factors suggested by Equation 7 look like for various desired levels of confidence $\alpha$ and tolerable error windows $\epsilon$. Table 4 shows various suggested scale factors when $\min (m,n) = 10$K, and Table 5 shows suggested scale factors when $\min (m,n) = 10$M. We notice that the theory accounts for a larger number of elements in the sets that are being compared against each other. With only 10K elements, if we want the estimated cosine to be within ± 7% (meaning $\epsilon = 0.07$) of the original cosine, then the theory suggests that we have to use a scale factor of 1. In other words, there is no scope for sub-sampling at this desired resolution. It is only at $\epsilon \ge 0.08$ that we can get away with sub-sampling, although the recommended scale factor is not very small to be drastically helpful in reducing computational resources.

**Table 6:** Fraction of times the estimated cosine falls within $\pm 5\%$ of the true cosine of *A* and *B*, for different sizes of *A* and *B*. The similarities were estimated **using a scale factor of 1/1000**, which is the default in sourmash. In a large fraction of times, the estimated cosine is *not* within $\pm 5\%$ of the true cosine

Num. elements in B	Num. elements in A				
	100K	200K	300K	400K	500K
100K	0.09	0.20	0.25	0.32	0.38
200K	0.17	0.29	0.55	0.47	0.51
300K	0.28	0.40	0.54	0.58	0.57
400K	0.32	0.45	0.61	0.71	0.74
500K	0.45	0.42	0.73	0.66	0.83

On the other hand, as documented in Table 5, we can use a scale factor of roughly $0.0006 \sim 0.0008$ to allow a 10% window for error when we have at least 10M elements. If we want to be *very* accurate and only allow 1% error, we need to obtain FracMinHash sketches with a scale factor of roughly $0.05\sim 0.08$. From Table 5, we also notice that with a higher level of desired confidence $\alpha$, we need to employ larger scale factors; although the effect a larger $\alpha$ has on the suggested scale factor is less prominent than the effect of a smaller $\epsilon$.

**Table 7:** Suggested scale factors for various $\min (|A|,|B|)$, as calculated by Equation 7. $\alpha = 0.95$, $\delta = 0.05$ was used

$\min (|A|,|B|)$	100K	200K	300K	400K	500K
Suggested scale factor	0.2414	0.1207	0.0805	0.0604	0.0483

We next show the usefulness of using these recommended scale factors, in contrast to a preset value. The state-of-the-art program to compute and analyze FracMinHash sketches is sourmash [6, 20], which uses a default scale factor of 1/1000. As a result, many studies that use sourmash use this default value, even though the tool can work with other non-default scale factors. We show that a preset scale factor may result in an error higher than expected. In this set of experiments, we simulated a universe of 1M elements. We then randomly selected two sets *A* and *B* from this universe. We varied the number of elements in these sets from 100K to 500K. The actual elements were selected randomly. We then calculated the true cosine of *A* and *B* using all elements. After that, we used the preset scale factor of 1/1000 to compute FracMinHash sketches of *A* and *B*, and estimated the cosine using these sketches. We also set the tolerable error rate ($\epsilon$) at 5%, and desired level of confidence ($\alpha$) at 95%. We then computed FracMinHash sketches of *A* and *B* using the scale factor suggested by Equation 7, and used these sketches to estimate the cosine. We then recorded if these estimated cosine values fall in the range $\cos \theta ~ (1 \pm 0.05)$. We repeated the experiment 1000 times for all sizes of *A* and *B*. Table 6 shows the fraction of times the similarities estimated using a fixed scale factor of 1/1000 fall within $\pm 5\%$ of the true cosine. Table 8 shows the fraction of times the similarities estimated using the scale factor recommended by Equation 7 fall within $\pm 5\%$ of the true cosine. The list of suggested scale factors in these scenarios is shown in Table 7.

**Table 8:** Fraction of times the estimated cosine falls within $\pm 5\%$ of the true cosine of *A* and *B*, for different sizes of *A* and *B*. The similarities were estimated **using the scale factor suggested by Equation **7. In almost all instances, the recommended scale factor can estimate the similarity so that the estimated value is within $\pm 5\%$ of the true similarity

Num. elements in B	Num. elements in A				
	100K	200K	300K	400K	500K
100K	0.99	1.0	1.0	1.0	1.0
200K	1.0	1.0	1.0	1.0	1.0
300K	1.0	1.0	1.0	1.0	1.0
400K	1.0	1.0	1.0	1.0	1.0
500K	1.0	1.0	1.0	1.0	1.0

These results show that 100% of times, the recommended scale factor can estimate a cosine within the tolerable error range, whereas using a preset scale factor can result in a larger error. Evidently, using the default scale factor of 1/1000 may not be well-suited where a higher resolution around the true value is required but the input sets are not sufficiently large.

### frac-kmc computes FracMinHash sketches faster

After establishing the conditions when FracMinHash sketches can be safely used to estimate the cosine similarity, we next wanted to use FracMinHash sketches on real biological sequences. Ideally, we wanted to show that by using FracMinHash sketches, we can compute the pairwise similarity matrix for a number of sequences faster than tools that use all $k$-mers. The fastest tool that can compute pairwise similarity/distance matrix from a list of sequences is currently Simka [2], whereas the state-of-the-art tool to compute FracMinHash sketches is sourmash [6, 33]. Naturally, we tried to use sourmash to first compute FracMinHash sketches, and later compare the sketches to obtain a pairwise similarity matrix. Unfortunately, we found that the command that computes FracMinHash sketches (called sourmash sketch) is many times slower than Simka. We noted that this is because sourmash treats input sequence files in a serialized manner, where there is scope for parallelism over multiple threads to make the processing faster.

**Fig. 1:**
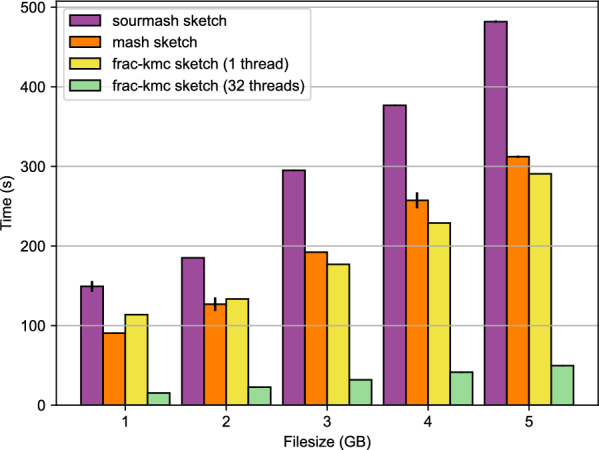
Wall-clock time required by the commands mash sketch, sourmash sketch and frac-kmc-sketch to compute a sketch. The input files are fastq.gz files containing metagenome samples taken from the human gut. MinHash sketches of 1000 was computed, and FracMinHash sketch with scale factors $s = 0.001$ was computed. When using 32 threads, frac-kmc finishes roughly 10 times faster than sourmash, and roughly 6.7 times faster than Mash. When running on a single thread, frac-kmc runs up to 70% faster than sourmash. On small inputs, frac-kmc runs roughly 20% slower than Mash, but runs roughly 15% faster for files larger than 5 GB

Therefore, for practical purposes, we decided to write a new FracMinHash sketch generator program by modifying a fast and efficient $k$-mer-counter KMC [22]. When KMC merges its $k$-mer bins, frac-kmc filters the $k$-mers and keeps only those that are below the acceptance threshold *Hs*, discarding others. Details of frac-kmc are included in Section Implementation of frac-kmc. We used gcc version 11.4.0 to compile frac-kmc and generate the following results.

Figure 1 shows a running time comparison for the commands sourmash sketch and frac-kmc sketch on files of different sizes. The files are fastq.gz files. These files were randomly selected from the Human Microbiome Project [32]. We verified that sketches produced by the two programs are identical by running sourmash compare. The comparison shows that frac-kmc consistently runs about 10 times faster than sourmash to sketch input files when run on 32 threads. When run on a single thread, frac-kmc runs roughly 30% faster on files of size 1 GB, and roughly 70% faster on files of size 5 GB. For this set of analyses, we used the latest version of sourmash: 4.8.8, as of 1 May 2024. Both tools were run to not keep track of abundances of $k$-mers. We ran the programs to compute sketches for $k = 21$ and scale factor $s = 1/1000$. We tested with other values of *k* and *s* and saw similar results.

Figure 1 also shows the average running time to compute MinHash sketches (sketch size = 1000 hashes) from the same files using Mash (version 2.0). On a single thread frac-kmc runs roughly 20% slower than Mash on files of size 1 GB, but runs roughly 15% faster for files larger than 5 GB. It is important to highlight that neither of Mash and sourmash is a parallel tool: neither of these two can run on multiple CPU-cores to speed up sketch generation from a single input file. Therefore, computing sketch from a very large file would take a long time if we use Mash or sourmash, which can be sped up if we use frac-kmc.

### frac-kmc estimates cosine similarity accurately

**Fig. 2:**
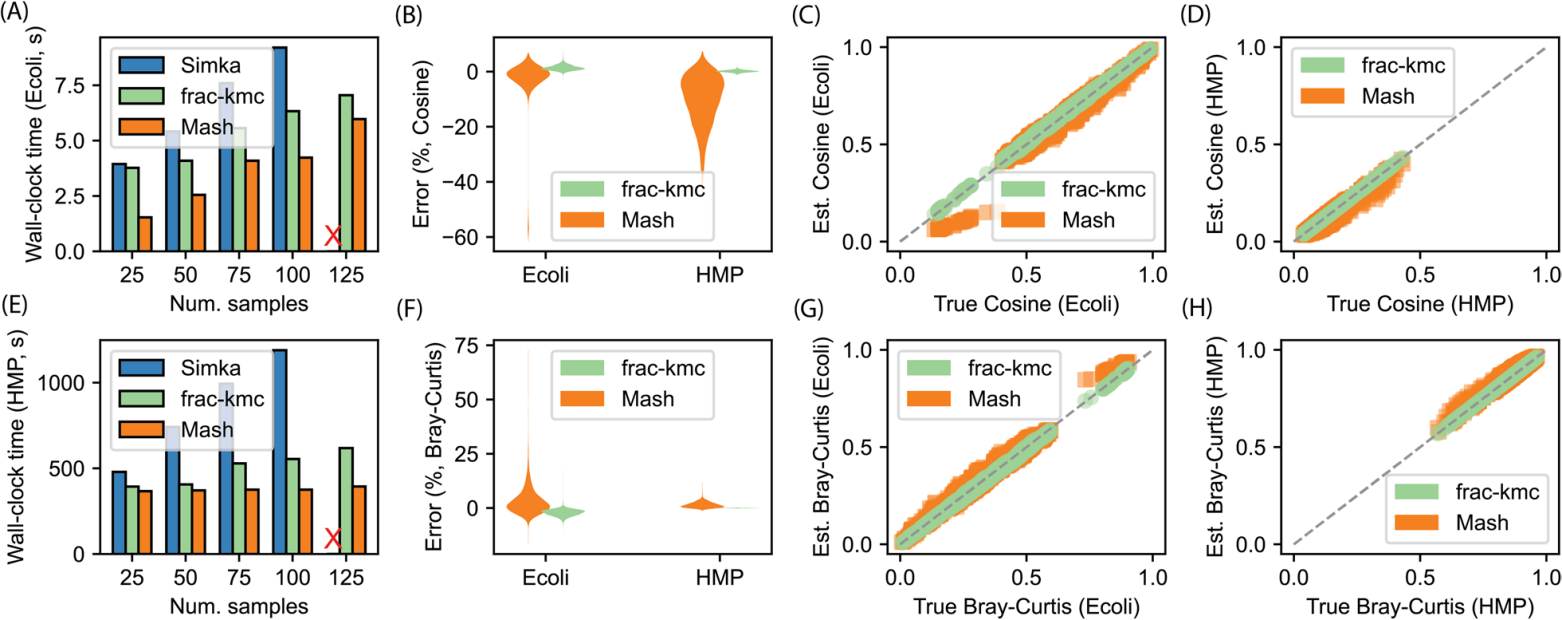
Running time and accuracy of the tools on **Ecoli** and **HMP** datasets.** A** and** E** show the total wall-clock time to run the tools for 25-125 randomly selected samples (Simka did not exit after > 48 hours).** B** and** F** show the distributions of the percentage errors when estimating cosine and Bray-Curtis, respectively, for 100 samples (ground truth was computed using Simka).** C** and** D** show estimated vs. true cosine values for the two datasets (100 samples), and** G** and **H** show the same plots for the Bray-Curtis dissimilarity. The plots show that frac-kmc is about 40-50% slower, but is comparatively more accurate in estimating the metrics

We next show that by using FracMinHash sketches computed by frac-kmc, we can estimate metrics faster than Simka [2] (which uses all $k$-mers to operate), and more accurately than Mash [31] (which uses fixed size MinHash sketches). For this set of experiments, we used two datasets: the **Ecoli** dataset contains 3682 E.coli genome assemblies, and the **HMP** dataset contains 300 metagenome samples randomly collected from the human gut, taken from the Human Microbiome Project [32]. We ran Simka, Mash, and frac-kmc on these datasets to compute the pairwise cosine similarity matrix and the pairwise Bray-Curtis dissimilarity matrix. We ran Simka and Mash by using a $k$-mer size of 21. We ran Mash to compute sketches of size 10K, and kept other settings at their default values. We used a scale factor $s = 0.001$ for the same $k$-mer size to compute FracMinHash sketches using frac-kmc. Details of the datasets, how the programs were run, and how the metrics were computed are elaborated in Section Generating results in Section 4.

Before presenting the results, it is important to note that these two datasets only allow genome-versus-genome and metagenome-versus-metagenome comparisons, but not genome-versus-metagenome comparisons. This is because MinHash sketches are shown to perform poorly when sets of very dissimilar sizes are compared, and therefore, for genome-versus-metagenome comparisons, MinHash sketches would naturally perform poorly, as shown previously [18]. Consequently, we only show comparisons among sets of similar sizes and use these two datasets.

Figure 2 shows the total wall-clock time required to run Simka, Mash, and frac-kmc to estimate the cosine similarity and the Bray-Curtis dissimilarity for 25-125 randomly selected samples. We found that as the number of samples reaches roughly 125, Simka does not exit even after letting it run for more than 48 hours. In addition to these extremes, we found that Simka operates by creating many SimkaCount and SimkaMerge processes, which are not spawned as descendants of the mother Simka process. Therefore, we found no good way to measure the CPU time/memory consumed by Simka, and are not including CPU time/memory for these smaller runs. Figure 2 also shows the accuracy of Mash and frac-kmc in estimating the metrics for the 100 samples in the form of the distributions of errors (B, F), and in the form of true-vs-estimated values (C, D, G, and H). The ground truth, in these cases, was the output of Simka. These results show that frac-kmc runs 40-50% slower compared to Mash, and offers a great improvement in accuracy.

**Fig. 3:**
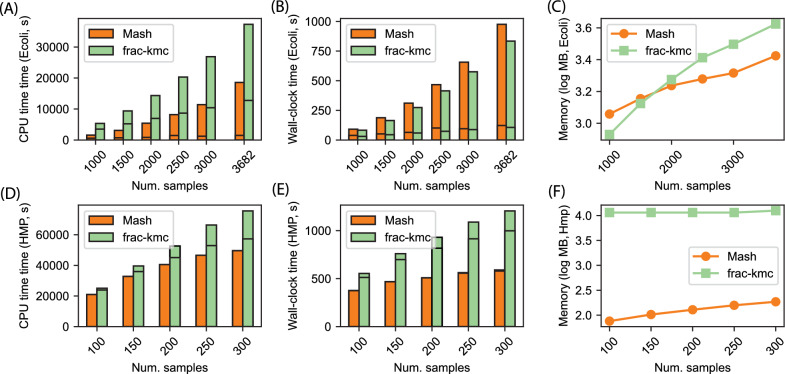
Computational resources consumed by Mash and frac-kmc when estimating metrics for a large number of samples. A, B, and C show the CPU time, wall-clock time, and peak memory usage for 1000-3682 samples in the **Ecoli** dataset.** D**,** E**, and** F** show the same plots for the **HMP** dataset, varying the number of samples from 100 to 300. Both tools were run using 128 threads. The bars in** A**,** B**,** D**, and** E** are split into two parts: the bottom part shows the time to compute the sketches only, and the top part shows the time to compute the metric from the sketches. Using frac-kmc is up to 18% faster and the memory usage is similar in the **Ecoli** dataset, where the samples are smaller genomes. On the other hand, frac-kmc runs about 2-2.1x slower in the **HMP** dataset (the samples are large metagenomes), and roughly uses 100x more memory than Mash. These heavier resource usages of frac-kmc allow for the highly accurate results shown in Figure 2

As shown in Figure 2, Simka does not scale beyond about 100 samples. We next investigate how Mash and frac-kmc scale for a larger number of samples. The resources used by these two tools are shown in Figure 3. The bars that show CPU time and wall-clock time are split into two parts: the bottom part shows the time required to only compute the sketches, and the top part shows the time to load the sketches into memory and estimate the metrics. For the **Ecoli** dataset where each sample is a relatively small genome, both MinHash and FracMinHash sketches are relatively small, and therefore, the wall-clock time and memory footprint are similar. The majority of the time, for both tools, is spent in computing the metrics, as there are a large number of pairs. We note here that Mash stores the sketches (for all the input samples) in a single binary file. To get individual sketches from this single file, we need to perform a mash dump program, which is the reason Mash is slightly slower than frac-kmc for the **Ecoli** dataset.

On the other hand, for the **HMP** dataset, the FracMinHash sketches computed by frac-kmc are much larger than MinHash sketches computed by Mash. As a result, frac-kmc runs about 2-2.1x slower than Mash. As frac-kmc produces larger sketches, the memory usage is also about 10GBs, compared to only 100MBs for Mash. These higher running time and memory usage allows frac-kmc to facilitate very accurate results, as shown in Figure 2B and F.

## Methods

### Implementation of frac-kmc

The core motivation behind implementing frac-kmc was that sourmash sketch dna was very slow for larger files. Therefore, we decided to use a fast and efficient $k$-mer-counting program. There are many $k$-mer-counters available in the literature, namely jellyfish [28], DSK [36], KMC [22] etc. We decided to use KMC since its source code was easy to understand and navigate. Instead of running KMC and iterating through all $k$-mers in KMC’s output, we decided to modify the source code so that only the $k$-mers in the sketch were retained in the output. This made the entire program many times faster since typical scale factors used to compute FracMinHash sketches are very small. Therefore, we implemented the 64-bit MurMurHash function in C++ within the source code of KMC, and made the necessary changes so that instead of keeping track of all the $k$-mers, the program now kept track of only the $k$-mers whose hash value fell below the cut-off threshold. As a result, the succinct $k$-mer-database constructed by this modified KMC now contained only the relevant $k$-mers. Finally, we modified the program kmc dump so that instead of writing all the *k*-mers in an output file, it now wrote the 64-bit MurMurHash values for the kmers in a sorted list – which is the output format of sourmash sketch. We named this program frac-kmc. After generating sketches from the same file using frac-kmc and sourmash, we used sourmash compare to confirm that the sketches are identical.

### Generating results in Section Experimental Results

#### Datasets

The datasets we used are:

1. *Ecoli* We collected all 3682 E. coli genome assemblies in NCBI.
2. *HMP* We collected whole genome shotgun sequences from the Human Microbiome Project [32]. We randomly selected 300 gzipped fastq files corresponding to samples collected from the human gut.

The metagenome samples in the **HMP** dataset have an average file size of 1.88 GB and a median file size of 1.72 GB. The smallest file size is 58 MB, and the largest file size is 5.5 GB. This dataset works as a stress test for all the tools, where the input files are very large, reflecting real-life metagenome data; although the number of total samples is manageable. On the other hand, the **Ecoli** dataset challenges all the tools because the number of samples is very large (there are roughly 67 million pairs), although every individual file is quite small and easy to process.

#### Running Simka, Mash, and frac-kmc

We ran Simka, Mash, and frac-kmc on the **Ecoli** and the **HMP** dataset, to produce the pairwise cosine similarity matrix and the pairwise Bray-Curtis dissimilarity matrix. Simka readily produces several similarity and dissimilarity metrics when invoked on a list of input files. However, it does not produce cosine similarity. Therefore, we took the Chord distances generated by Simka and converted them to cosine similarities.

We used Mash and frac-kmc to compute MinHash and FracMinHash sketches of the input files, respectively. We then used a parallelized program to read all the sketches and compute cosine and Bray-Curtis using the sketches. We ran Mash to generate MinHash sketches of size 10,000, which is 10x the default value. The minimum number of $k$-mers in all files we used was roughly 4.8 million. In such a case, the minimum scale factor suggested by Equation 7 is 0.0005 (using $\epsilon =10\%, \alpha = 0.95$). Therefore, we simply used the sourmash default value, 1/1000 to generate the FracMinHash sketches when running frac-kmc. All three tools were run on 128 cores of the same machine, including the multi-threaded code segment that reads MinHash and FracMinHash sketches, computes pairwise cosine similarity values, and writes them into an output file. When we invoked Mash, we provided Mash with all files at once using a file list, and used the option-p to use 128 threads. When running frac-kmc, we did not use a file list, but spawned a different frac-kmc process for every input file.

### Proofs of theorems

#### Theorem 1

Let $\Omega = \{~e_i~\}_{i=1}^N$ be a given set (universe), and let $A \subseteq \Omega$. Let $\textbf{FRAC}_{s}(A)$ be the FracMinHash sketch of *A* for a given *s* where $0 \le s \le 1$, and let the cardinality of $\textbf{FRAC}_{s}(A)$ be $X_A$. The expected number of elements in $\textbf{FRAC}_{s}(A)$ is given by the following:

$$\begin{aligned} E\Big [ X_A \Big ] = ~s ~ \vert A \vert . \end{aligned}$$

#### Proof

Let $I_i$ be an indicator variable as follows:

$$\begin{aligned} I_i(e_i) = {\left\{ \begin{array}{ll} 1 & \text { if } e_i \in \textbf{FRAC}_s(A) \\ 0 & \text { otherwise} \end{array}\right. } \end{aligned}$$

for all *i* such that $e_i \in A$. Using the uniformity property of the hash family we consider in this work and taking expectations over this hash family, $E[ I_i ] = \Pr [ I_i = 1 ] = s$.

Using these facts, we have the following:

$$\begin{aligned} E[ X_A ] = \sum _{i:e_i \in A} E[I_i] = \sum _{i:e_i \in A} s = s~|A|. \end{aligned}$$

$$\square$$

#### Theorem 2

Let $\Omega = \{~e_i~\}_{i=1}^N$ be a given set (universe), and let $A \subseteq \Omega$. If $\textbf{FRAC}_{s}(A)$ is the FracMinHash sketch of *A* for a given *s* where $0 \le s \le 1$, and if the cardinality of $\textbf{FRAC}_{s}(A)$ is $X_A$, then the following holds for any $\epsilon$ where $0< \epsilon < 1$.

$$\begin{aligned} \Pr \Bigg [ \Big \vert X_A - s ~ |A| ~\Big | \ge \epsilon ~ s ~ |A| ~\Bigg ] \le 2 \exp { \Big ( - s ~ |A| ~ \epsilon ^2/3 \Big ) }. \end{aligned}$$

#### Proof

Using the same indicator $I_i$ used in the proof of Theorem 1, we note the following:

$$X_A = \sum _{i:e_i \in A} I_i~~.$$

Using the independence property of the hash family, we note that all $I_i$ are independent. Therefore, $X_A$ is simply a sum of independent Bernoulli random variables. This allows for the use of the Chernoff concentration inequality (introduced in Section Preliminaries) for $\sum _{i:e_i \in A} I_i$, which completes the proof. $\square$

#### Theorem 3

Let $\Omega = \{~e_i~\}_{i=1}^N$ be a given set (universe), and let $A, B \subseteq \Omega$ be two sets in the universe. Let the cosine similarity of the sets *A* and *B* be $\cos \theta$, and that of the sets $\textbf{FRAC}_{s}(A)$ and $\textbf{FRAC}_{s}(B)$ be $\cos \theta '$, where $\textbf{FRAC}_{s}(A)$ and $\textbf{FRAC}_{s}(B)$ are the FracMinHash sketches of *A* and *B* respectively for a given *s* where $0 \le s \le 1$ Then, there exists an $\epsilon$ where $0< \epsilon < 1$, such that the following holds.

$$\begin{aligned} \Big \vert \cos \theta ' - \cos \theta \Big \vert \le \epsilon ~ \cos \theta \end{aligned}$$

with a probability of at least $1 - 6 \exp \Big \{-s ~ |~A\cap B~| ~\epsilon ^2 / ~[3(2+\epsilon )^2]\Big \}$.

#### Proof

As $\cos \theta '$ is estimated using $\textbf{FRAC}_{s}(A)$ and $\textbf{FRAC}_{s}(B)$, we have the following.

$$\begin{aligned} \cos \theta ' = \frac{|\textbf{FRAC}_{s}(A) \cap \textbf{FRAC}_{s}(A)|}{\sqrt{ |\textbf{FRAC}_{s}(A)| \times |\textbf{FRAC}_{s}(B)| }} \end{aligned}$$

For notational simplicity, let $X_S$ be the cardinality of the set $\textbf{FRAC}_{s}(S)$. Therefore, we have the following.

$$\begin{aligned} \cos \theta ' = \frac{X_{A\cap B}}{\sqrt{X_A ~ X_B}} \end{aligned}$$

By applying the results in Theorem 2 for the sets *A*, *B*, and $A \cap B$, we have the following results:

8 $$\begin{aligned} (1-\epsilon ) ~s ~|A|&\le ~~~~X_A~~~ \le (1+\epsilon ) ~s ~|A|~~, \end{aligned}$$

9 $$\begin{aligned} (1-\epsilon ) ~s ~|B|&\le ~~~~X_B~~~ \le (1+\epsilon ) ~s ~|B|~~, ~~\text {and} \end{aligned}$$

10 $$\begin{aligned} (1-\epsilon ) ~s ~|A\cap B|&\le ~~X_{A\cap B}~ \le (1+\epsilon ) ~s ~|A \cap B|~~. \end{aligned}$$

The probability with which these three results hold are at least $1 - 2 e^{-s|A|\epsilon ^2/3}$, $1 - 2 e^{-s|B|\epsilon ^2/3}$, and $1 - 2 e^{-s|A\cap B|\epsilon ^2/3}$, respectively, for $0 \le \epsilon < 1$.

By dividing Equation 10 by the square root of Equation 8 and Equation 9, we have the following:

$$\frac{{1 - \in }}{{1 + \in }}~\frac{{|A \cap B|}}{{\sqrt {|A| \times |B|} }} \le ~~\frac{{X_{{A \cap B}} }}{{\sqrt {X_{A} ~X_{B} } }}~~ \le \frac{{1 + \in }}{{1 - \in }}~\frac{{|A \cap B|}}{{\sqrt {|A| \times |B|} }} \Rightarrow \frac{{1 - \in }}{{1 + \in }}~\cos \theta \le ~~\cos \theta ^{\prime}~~ \le \frac{{1 + \in }}{{1 - \in }}~\cos \theta {\text{ }}$$

The probability with which this expression holds is at least $1 - 2 e^{-s|A|\epsilon ^2/3} - 2 e^{-s|B|\epsilon ^2/3} - 2 e^{-s|A\cap B|\epsilon ^2/3}$, which can be obtained by taking a union bound on the probabilities associated with Equations 8 through 10.

We next note that if $\frac{1+\epsilon }{1-\epsilon } = 1 + \delta$, then $\epsilon = \frac{\delta }{2+\delta }$. Also, if $\frac{1-\epsilon }{1+\epsilon } = 1 - \delta$, then $\epsilon = \frac{\delta }{2-\delta }$. To facilitate the stricter condition, we use the smaller value of $\epsilon$ given a $\delta$, which is $\frac{\delta }{2+\delta }$. Therefore, we argue that the following holds:

$$\begin{aligned} (1-\delta ) \cos \theta \le ~\cos \theta '~ \le (1+\delta ) \cos \theta , \end{aligned}$$

with a probability of at least $1 - 2 e^{-s|A|\epsilon ^2/3} - 2 e^{-s|B|\epsilon ^2/3} - 2 e^{-s|A\cap B|\epsilon ^2/3}$, where $\epsilon = \frac{\delta }{2+\delta }$. Using the fact that $\min \Big \{ |A|, ~|B|, ~|A \cap B| \Big \} = |A \cap B|$ completes the proof. $\square$

## Discussions

### Conclusions

Sketching-based methods allow practitioners to lower computational resource usage many-fold while keeping the accuracy reasonably well. In this paper, we analyzed such a sketching technique, FracMinHash, in estimating an array of similarity and distance metrics. We analyzed the conditions when it is theoretically sound to use FracMinHash and estimate these metrics, and suggested a minimum scale factor that is safe to use. We also presented a fast FracMinHash sketch generator tool frac-kmc and benchmarked its running time against Simka and Mash. We found that when a huge number of small samples are compared, using frac-kmc is nearly as fast as Mash in wall-clock time. When a number of larger samples are compared, using frac-kmc requires more time, although the results produced by frac-kmc are more accurate and precise. Our analyses show that when very large sequence files need to be sketched using FracMinHash, using frac-kmc can be especially useful.

### Further improvements

From a theoretical point of view, we can next study the behavior of other metrics that cannot be expressed as ratio of sizes of sets *A*, *B*, $A \cap B$, etc. Examples of such metrics are the Chord distance, the Hellinger distance, and the Jensen-Shannon distance. Although these metrics do not follow an agreeable mathematical form, experiments as well as the law of large numbers suggest that for these, too, we can prove nice asymptotic behaviors. From an implementation perspective: the programs we used may be improved and extended in several ways: the code we used to read in MinHash and FracMinHash sketches (generated by Mash and frac-kmc) is written completely in Python, without a particular focus on optimization. A well-written C++ implementation may improve things further. The implementation of MurMurHash64 in frac-kmc makes use of C++ optimizations, although we did not explore if they can be improved further. frac-kmc currently does not support protein $k$-mers (which sourmash does). Instead of using an exact $k$-mer-counter, other approximation-based inexact $k$-mer-counter program may be explored. And finally, sketches of minimizers (instead of all $k$-mers) may be employed to investigate even more aggressive downsampling.

## Data Availability

No datasets were generated or analysed during the current study.

## Footnotes

1 Strictly speaking, for the form of the Chernoff bound we use in the following, a fully mutually independent family of hash functions would be required, not just 2-wise independent. It is straightforward but not particularly illuminating to extend this hash family definition. Additionally, it is well known that such Chernoff-Hoeffding bounds hold for *k*-wise independent families for any *k* [37, Theorem 1], with small, but intricate constant factor differences, and it is convention in the sketching literature to use the “cleaner” Chernoff bounds we use below.
